# Electrophoresis assisted time-of-flow mass spectrometry using hollow nanomechanical resonators

**DOI:** 10.1038/s41598-017-03846-y

**Published:** 2017-06-14

**Authors:** Swathi Chaudhari, Kamalesh Chaudhari, Seokbeom Kim, Faheem Khan, Jungchul Lee, Thomas Thundat

**Affiliations:** 1 Department of Chemical and Materials Engineering, University of Alberta, Edmonton, Alberta T6G 2V4 Canada; 2 Department of Mechanical Engineering, 35 Baekbeom-ro (Sinsu-dong), Mapo-gu, Sogang University, Seoul, 04107 Korea

## Abstract

This report discusses the first demonstration of electrophoresis assisted time-of-flow mass spectrometry using ‘U’ shaped hollow nanomechanical resonators (HNR). Capillary electrophoresis was coupled with the HNR based mass detection to overcome low ionic conductivity of channels embedded in the HNR preventing direct *in-situ* electrophoretic separation. The flow of analytes through the HNR was achieved by balancing the hydrodynamic pressure to override the electromotive force and inhibit the motion of analytes towards the anode for capillary electrophoresis. The resonance frequency shifts of the HNR vibrating around 1.5 MHz were correlated with the time of the passage of the protein bands to construct the mass spectrum. The proposed concept was demonstrated by constructing a mass spectrum of egg white proteins in the molecular weight range of 14–250 kDa. When compared to regular polyacrylamide gel electrophoresis, our method not only provides a precise and fast readout but also avoids the use of chemical staining. This study paves a new route for low-cost and on-chip mass spectrometers with ultra-miniaturized dimensions.

## Introduction

High-resolution mass sensing capability of nanomechanical resonators has been demonstrated in recent years^1–4^. High frequency resonators operating in a vacuum with high quality factors have enabled sub-femtogram mass resolution for biomolecules, single cells as well as single nanoparticles^5^. While resonators fabricated by top-down silicon based micro- and nano fabrication techniques have shown ultra-high mass resolution, resonators fabricated with bottom-up synthesized carbon based nanomaterials have demonstrated further improved mass sensing with atomic resolutions^2, 4, 6^. It has been envisioned that nanomechanical resonators may enable single-proton resolution mass detection in the near future^5, 7^. One of the major benefits of nanomechanical mass spectrometers is that molecules need not be ionized prior to the detection of their masses^8^. However, such nanomechanical resonators used for mass sensing are based on the adsorption of analytes on their surfaces which require the whole system to be inside vacuum to avoid interference from airborne molecules^9, 10^. This condition makes it difficult to analyze samples that need to be maintained in solution phase during measurements. Hence, among many different types of resonators, hollow channel resonators (HCR) are emerging as a potential platform for measuring minute changes in the properties of liquid flowing through integrated channels with exceptional responsivity^5, 11–13^. Since liquid filled HCRs are easily interfaced with on- or off-chip vacuum, they enable high-resolution mass measurements for analytes in liquid, especially biological molecules.

The HCR platform allows sensing of analyte properties in its native environment and has been used for sensing the mass, volume, and density^14^ of cancer cells in physiological conditions^13^. Furthermore, mass sensing ability of nanochannel resonators has been used for attogram scale measurements of single nanoparticles in solution^3, 7^. However, when similar measurements need to be performed for comparatively lower weight molecules, mass resolution of all HCR reported to date is yet limited to ensemble rather than single molecules^5^. For molecular detection, suspended microchannel resonators (SMR), one of the HCR devices pioneered by Manalis group, have been retrofitted to realize on-chip pre-concentration and subsequent sensing of enriched molecules^15^. In the case of a mixture of molecules with unknown concentrations, separation of molecules based on size or charge becomes crucial.

For solution phase analysis with HCR, molecules need to be properly separated and introduced into integrated channels of HCR sequentially. A highly sensitive SMR was coupled with high-performance liquid chromatography (HPLC) to detect the eluted sample from the separation column with femtogram level mass resolution^16^. Although this demonstration was very promising and encouraging, a bulky and high-end HPLC setup used can hardly be adopted for portable field applications. Moreover, the performance of HCR has been greatly improved by downsizing the resonator since then^3^, and there are no follow-up attempts after the aforementioned work by Son *et al*.^16^. Hollow nanomechanical resonators (HNR) recently developed via silicon self-assembly in our group^17^ would be ideal once they are combined with a separation technique that requires minimum external setup. Towards this goal, we have developed a simple standalone system that can be packaged into a portable platform. While designing such a device, separation of molecules directly through the HNR was a challenge since the channel dimensions integrated in the resonator are too small for stationary phase of gels or separation columns to be loaded. In addition, ultra-low ionic conductivity of integrated channels makes it difficult to realize charge-based separation of mixed molecules even at the time scale of several hours. Moreover, such a low ionic conductivity results in temperature rise in channel that can also affect the resonance frequency of the HNR. To overcome the aforementioned issues, a novel method has been developed in which a mixture of protein molecules was separated by capillary gel electrophoresis. Separated protein bands were then passed through integrated channels of the resonator by balancing the hydrodynamic pressure so as to override an electromotive force towards the anode of capillary electrophoresis.

## Results

The HNRs used in this study were ‘U’ shaped cantilevers with 34 µm in length and 16.5 µm in width. The width, height, and wall thickness of channel were 4.5, 1.4, and 0.3 μm, respectively. Briefly, the HNR devices were fabricated by a series of conventional microfabrication approaches which involves self-assembled cavity formation upon high temperature annealing, cavity internal oxidation, and device release via surface micromachining. A double-sided, polished (100) silicon wafer was anisotropically etched by deep reactive ion etching (DRIE) and annealed at 1,150 °C for 24 min under argon atmosphere for cavity formation by silicon self-assembly. Then, the interiors of cavities were dry-oxidized to define a thin wall of resonator and to acquire etch selectivity over the surrounding silicon during the device release to be followed. Oxide tube resonators were released by a combination of DRIE and SF_6_ etching to minimize undercuts. Finally, suspended HNRs were covered with a glass wafer and diced into single chips which could be individually mounted and used in a custom vacuum holder^17^ offering off-chip vacuum of ~10^−5^ Torr with integrated microfluidic feed-throughs.

**Figure 1:**
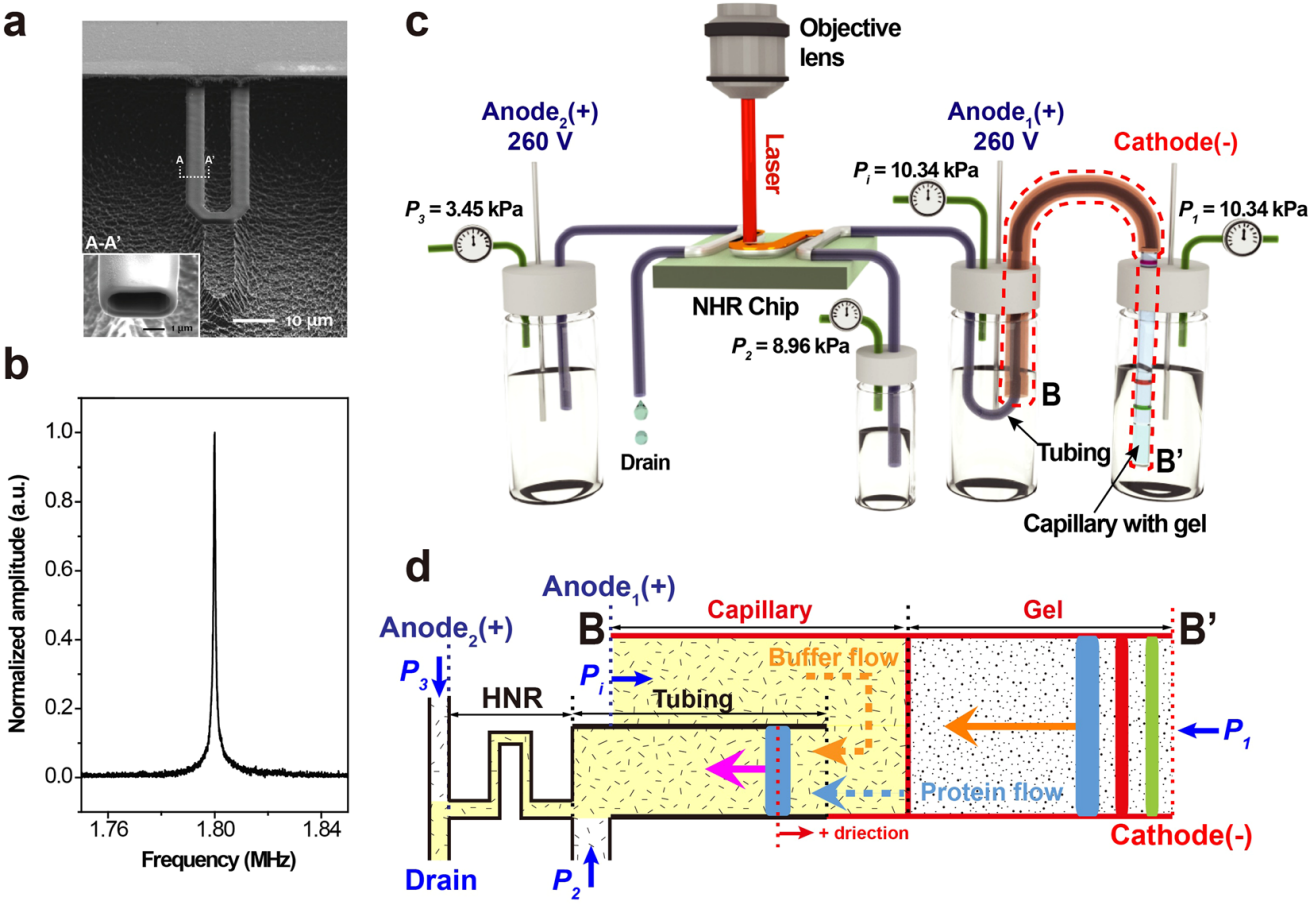
Schematic and explanation of electrophoresis assisted time-of-flow mass spectrometry setup. (**a**) Scanning electron micrograph (SEM) of the HNR used in this study. Scale bar is 10 µm. Inset shows SEM of the cross-section of the integrated channel in the HNR which is cut along A-A′ by focused ion beam milling. Scale bar is 1 µm. (**b**) Amplitude spectrum of the HNR in (**a**) showing the resonance frequency of 1.8 MHz in vacuum. (**c**) Simplified representation of the experimental setup used for the proposed electrophoresis assisted time-of-flow mass spectrometry using HNR. The setup consists of a pressure controlled sample delivery, bias electrodes for electrophoretic separation, and laser based resonance detection. One of sample access ports of HNR is connected to the output of capillary where electrophoretic separation takes place. (**d**) The region enclosed a dashed contour (BB’) in (**c**) is zoomed in and simplified to explain the working principle of electrophoresis assisted time-of-flow mass spectrometer.

Figure 1a shows a scanning electron micrograph of the fabricated HNR. The resonance frequency and quality factor of the HNR shown in Fig. 1a are 1.8 MHz and 2,500 in vacuum, respectively, measured by using a laser Doppler vibrometer (LDV, MSA-500, Polytec) (Fig. 1b). The HeNe laser in the LDV was focused on to the free end of the HNR through a 20X objective. To be used as a time-of-flow mass detector, the mass density responsivity of the HNR was acquired by introducing ethanol-water binary mixtures (Supplementary Information Fig. S1). The resonance frequency of the HNR linearly decreased with the mixture density and the density responsivity of −238 Hz kg^−1^ m^3^ was obtained from the linear regression of the frequency vs. density calibration.

For mass spectrometric measurements, poly-acrylamide gel electrophoresis was performed in a glass capillary tube with the inner diameter and wall thickness of 1.2 and 0.2 mm, respectively. The capillary tube was filled with stacking gel of 0.5 cm (6%) followed by resolving gel of 3 cm (12%). Separation of egg white proteins was carried out electrophoretically in the presence of 2X Tris running buffer. Separated proteins were transported using a Teflon tube (the inner and outer diameters of 230 µm and 800 μm, respectively) connected to the HNR input channel (Fig. 1c). This provides a radius difference of ~200 µm between the outer wall of the Teflon tube and the inner wall of the capillary tube which allows sufficient ionic current necessary for electrophoresis. Anode_1_, anode_2_, and cathode made of 1-mm diameter copper wires were connected to two power supplies (N5770A, Keysight) in series configuration to apply the electric potential difference required. As shown in Fig. 1c, a potential difference of 260 V was applied between the anode_1_ and the cathode, with the cathode placed at the sample loading side of the capillary tube. This facilitates electrophoretic separation to take place across the capillary tube. The same potential difference of 260 V was also applied between anode_2_ and the cathode to equalize potentials at the input and output channels of the HNR so as to avoid further spreading of charges, once they come out of the gel and enter the HNR channel. At the same time, pneumatic pressures of *P*_*i*_ and *P*_*2*_ were applied at the input of the HNR to facilitate the passage of separated proteins through the HNR. Of note, *P*_*2*_ was kept slightly lower than *P*_*i*_ to avoid the backflow of proteins towards anode_1_. In the absence of potential at anode_2_ or pressures (*P*_*i*_ and *P*_*2*_), separated protein bands do not enter the HNR, and thus, no shift in the HNR resonance frequency was observed. In fact, the applied pneumatic pressures expedite the proposed time-of-flow mass spectrometry. During the experiment, pressures applied at various locations have been kept constant using electro-pneumatic pressure regulators (ITV2030-312CL5, SMC). Therefore, the hydrodynamic velocity across the HNR remains constant thus more consistent time-of-flow observations are enabled. Flow of protein molecules through the HNR is possible in our setup because of the applied pressure difference. These flowing charges then complete the circuit at anode_2_. Such pressure-controlled charge flow manipulations have been previously used in ion concentration polarization devices, designed for the separation of molecules or impurities from water^18, 19^.

For detailed explanation, a simplified schematic of the complete setup is shown in Fig. 1d. From the directions of pressure-controlled flow at the HNR-capillary junction (BB′) and through the HNR channels, it can be understood how charges can also complete the circuit at the drain electrode (anode_2_). Pressure at both ends of the capillary tubes (*P*_*i*_ and *P*_*1*_) were maintained equal to avoid movement or rupture of the gel column during electrophoresis. In addition, due to high viscosity of the gel column, pressure is almost ineffective on the molecules inside the gel where electromotive force becomes predominant. The velocity of the proteins in the gel medium (*v*_*Capillary*_) can be estimated by Stokes equation:

1 $${v}_{Capillary}={u}_{ep}E=\frac{q}{6\pi \eta r}E$$

where *u*_*ep*_ is the electrophoretic mobility, *q* is the charge of the protein, *η* is the dynamic viscosity of the media, *r* is the radius of the protein, and *E* is the applied electric field.

Once the protein molecules come out from the gel medium into the buffer medium, hydrodynamic force due to the applied pressure overrides the electrophoretic and electro-osmotic forces. Additionally, the velocity of the proteins flowing through the HNR is a function of the pressure gradient across the HNR as well as streaming current. The later occurs, mainly because of the electric field created by depletion of positive and negative charges at both ends of the HNR. The streaming current exists in opposite direction and remains much weaker than the pressure driven flow, therefore it can be ignored. Ultimately, the average velocity of the proteins (*v*_*HNR*_) can be explained through Hagen-Poiseuille Equation.

2 $${v}_{HNR}=\frac{{\Delta }P{R}^{2}}{8\eta L}$$

where Δ*P* = −(*P*_*i*_ + *P*_*2*_ − *P*_*3*_)/*2* is a pressure difference across the HNR, *R* is the radius of the integrated channel (of the HNR), *η* is dynamic viscosity of the media, and *L* is length of the HNR channel. *ΔP* is negative to induce the hydrodynamic flow towards the drain. If *ΔP* is positive, the separated protein bands would not flow through the HNR without applying unreasonably high electric potential. In addition, if *ΔP* is a large negative value, the separated protein bands may overlap thus prevent the HNR from capturing well-separated peaks with the limited measurement speed of the LDV and fluidic interfaces such as tubing and sealing gaskets may partially fail and ultimately result in leakages. Considering these aspects, applied pressures were determined.

To establish the proposed concept, a sample of egg white was used. The composition of egg white allows analysis of proteins over wide range of molecular weights as well as concentrations. With hydrodynamic pressures satisfying the condition in equation (2), separated protein molecules in the single band corresponding to a specific mass-to-charge ratio enter the HNR. When these single bands sequentially pass through the HNR channel, corresponding density changes are reflected as changes in the resonance frequency of the HNR. Variations in the resonance frequencies can be temporally correlated with the molecular weight of proteins to construct a mass spectrum of the separated proteins.

**Figure 2:**
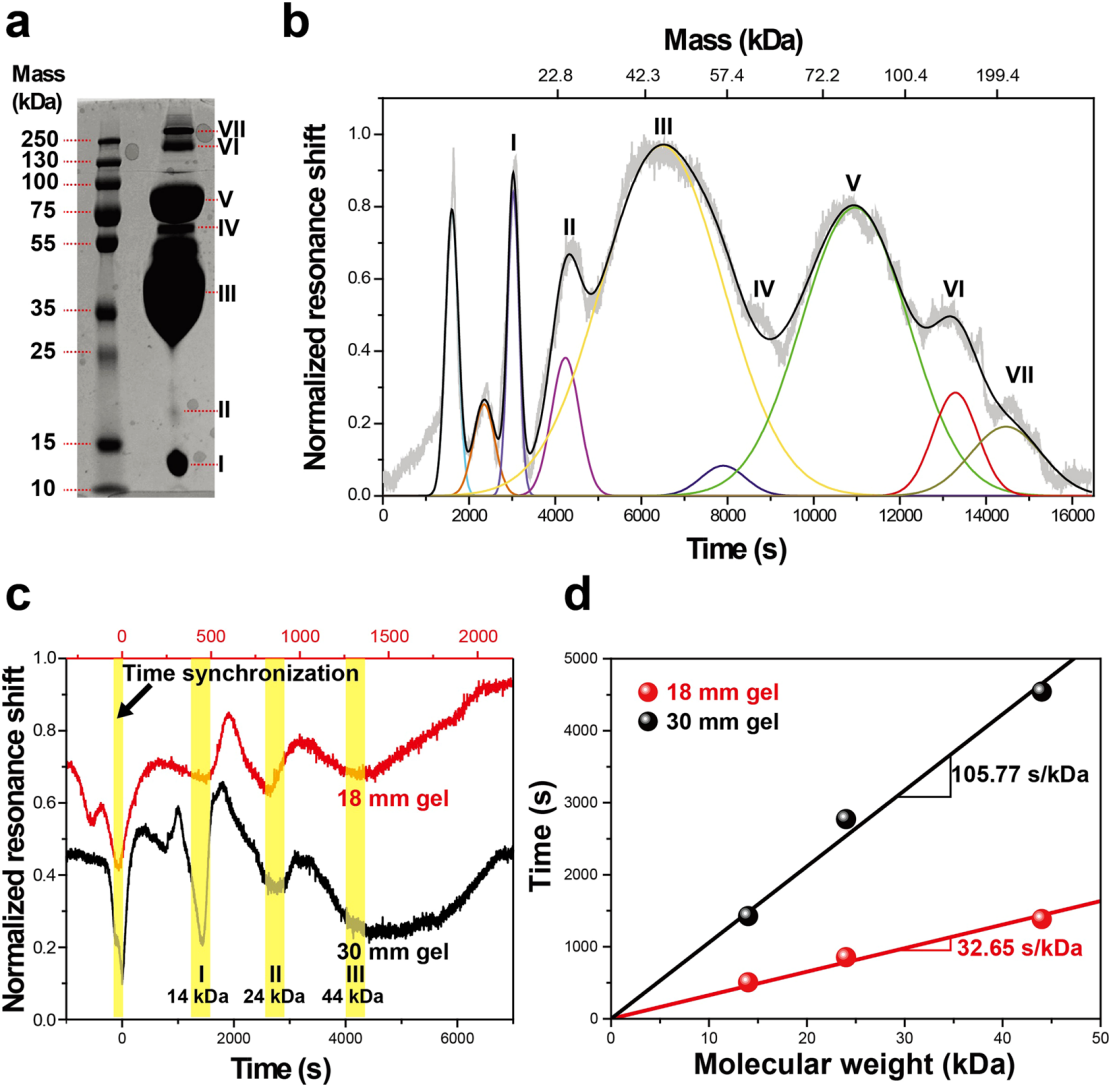
Comparison of PAGE and Time-of-flow mass spectrum of egg white proteins. (**a**) Polyacrylamide gel electrophoresis (PAGE) of egg white sample is shown along with the PAGE standard in the molecular weight range of 10–250 kDa. **(b**) Time-of-flow mass spectrum of egg white. Peaks below certain area value have been neglected as noise. Mass scale on top is shown only for the region 14–250 kDa as only this mass range has been verified using PAGE. (**c**) Comparison between time-of-flow mass spectra and (**d**) rate of protein elution in two different gel lengths (18 and 30 mm).

To validate the aforementioned concept, regular polyacrylamide gel electrophoresis of egg white sample was performed. The bands obtained for egg white are shown in Fig. 2a. These protein bands were correlated with the changes in the resonance frequency of the HNR to obtain mass vs. time calibration curve for the mass spectrometry. The observed shift in the resonance frequency may not correlate with the actual molecular weight of the proteins since the HNR measures the changes in the density of the solution passing through it. Therefore, although the electrophoretic bands are composed of the molecules with different molecular weights, the final molar concentration of the flowing sample may change due to the spread of bands. By using protein samples of known molar concentrations, it has been shown that the shift in the resonance frequency of the HNR exhibits excellent linearity with the concentration of protein (Supplementary Information Fig. S2). The responsivity of the HNR to changing molar concentrations of bovine serum albumin was found to be −4.2947 Hz µM^−1^ in deionized water and −1.8064 Hz µM^−1^ in Tris buffer, respectively. Hence, similar to conventional mass spectrometric techniques, which use time-of-flight analysis to construct the mass spectrum of analytes, time-of-flow analysis have been used in this study. The mass spectrum constructed using the raw data (Supplementary Information Fig. S3) obtained from the changes in the resonance frequency of the HNR is shown in Fig. 2b. This shows that the mass spectrum starts with two peaks, which are unaccounted for proteins present in the sample. These peaks were caused by the dye front which can be observed when analysis was carried out in the absence of proteins in the sample (Supplementary Information Fig. S4). The peaks, which appear after first two peaks, are for the proteins present in the sample. This can be seen by direct comparison between the bands for egg white in Fig. 2a and b. We have also compared our results with the mass spectrum obtained from matrix assisted laser desorption ionization (MALDI) mass spectrometry (Supplementary Fig. S5). Due to the complexity of egg white composition and appearance of multiple charges and dimers, we have used only major peaks in the MALDI mass spectrum. For a better estimation of peak positions and corresponding parameters in time-of-flow mass spectrum, the temporal resonance frequency shift curve (not to be confused with a resonance frequency curve which is a Lorentzian) was normalized and fitted with the Gaussian distribution function. Details of the molecular weight and percentage abundance of major proteins in egg white are provided in Supplementary Information Table S1.

To check the system-dependent variations, two different gel lengths (18 and 30 mm) for capillary gel electrophoresis were used. Fig. 2c shows time-of-flow mass spectra in different gel lengths. Time in measurements was synchronized by a dye indicator. Then, 14, 24, and 44 kDa proteins in the egg white sample were similarly detected. Fig. 2d shows the rate of protein elution in different gel lengths. The linear fitted protein elution rates are 32.65 and 105.77 s/kDa in 18 and 30 mm, respectively. The longer gel took the longer time due to the higher flow resistance in the gel.

**Figure 3:**
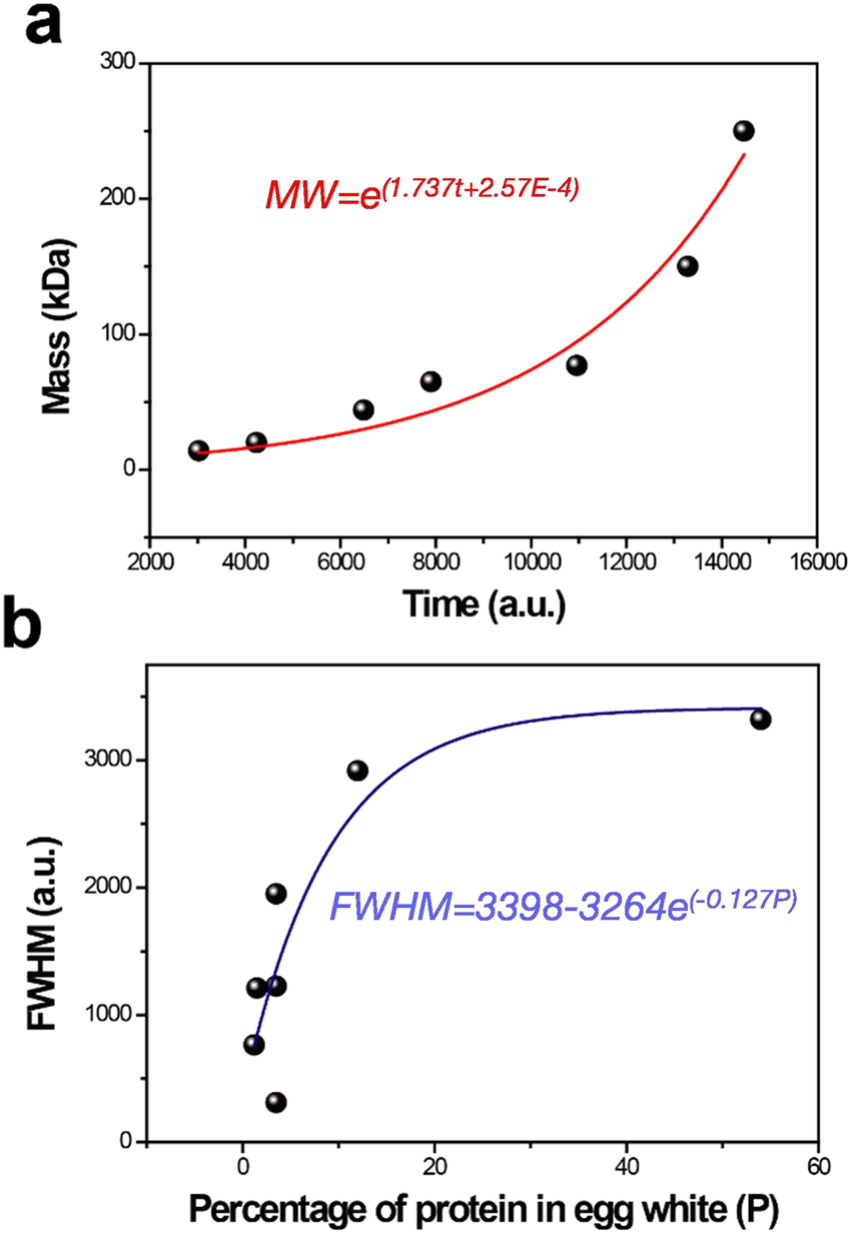
Calibration curve for the time-of-flow mass spectrometry. (**a**) The calibration curve obtained from the fitted peak positions and molecular weight of the proteins. **(b)** Variations in the FWHM of peaks in mass spectrum with respect to the percentage of proteins present in egg white sample.

The calibration curve obtained from the fitted peak positions and molecular weight of the proteins is shown in Fig. 3a. In electrophoresis, the natural logarithm of molecular weight exhibits linear relationship with the electrophoretic mobility of a protein in the buffer, which can be calculated as the ratio of the distance migrated by protein molecules to the distance migrated by the dye-front^20^. Hence the equation to correlate molecular weight and electrophoretic mobility can be expressed by equation (3).

3 $$\mathrm{ln}(MW)=a{\mu }_{ep,buffer}+b$$

where *MW* represents the molecular weight of a protein. The quantities, *a* and *b* are the slope and intercept of the line, respectively. In the present experiment, considering the elution time of a particular protein band is directly proportional to the electrophoretic mobility, the calibration curve can be fitted using equation (4).

4 $$MW={e}^{(a^{\prime} t+b^{\prime} )}$$

where *t* is the time of the passage of a protein through the HNR, *a*′ and *b*′ are the slope and intercept of the fitted line, respectively.

Additionally, it was observed that, although the amount of shift in the resonance frequency cannot be exactly correlated with the protein concentration, the full width at half maximum (FWHM) of the corresponding Gaussian distribution function provides a quantitative estimate of a protein present in the mixture of proteins (Fig. 3b). In the lower molecular weight regime, the mass vs. time curve is almost linear, but for higher molecular weight proteins, time resolution is limited due to their low mobility. Time resolution also depends on the difference between molecular weights of the proteins. Proteins with nearly identical molecular weights cannot be discerned from the mass spectrum as their bands tend to merge. Our experiments suggest the ability of our time-of-flow mass spectrometry to resolve proteins with the molecular weight difference of ~10 kDa.

## Discussion

A technique has been developed which is ideal for miniaturization of the standalone systems required for mass spectrometric analysis in liquid media. We would like to mention that, although herein we have provided promising proof-of-concept measurements, we have also observed system dependent variations in the time-of-flow mass measurements using the HNR (Supplementary Figure S6). Considering the preliminary nature of the setup, such variations can be attributed to the changes in gel length in capillary, imperfection in capillary-HNR coupling, etc. Without a doubt, the mass resolution as well as the speed of the analysis by electrophoretic techniques can be further improved by optimization in components and system integration, which can efficiently couple the microfluidic electrophoresis with HNR detectors. The technique presented here can be used for mass analysis in the range 10–250 kDa which indirectly improves the applicability of the HNR as compared to other HCRs which may sense either the weight of single solution phase analytes with molecular weight above 600 kDa^3, 7^ or analytes below this molecular weight in vacuum^9^. Another benefit of the technique is that the HNR devices used in this study are fabricated using silicon self-assembly and post oxidation, which can provide a device made of silicon dioxide. Silicon dioxide is preferred over silicon for the electro-osmotic operation necessary for carrying out capillary electrophoresis. These aspects allow miniaturization of the device down to true nanoscale. Hence, it will be easy to combine the capillary electrophoresis setup used in the present study with the HNRs. The present study also opens up the possibility of further investigations of this setup for complete formulation and quantitative description of the experimental parameters by appropriate simulations. We believe that the presented technique will be of great importance in the development of portable mass spectrometers for field use.

## Methods

### Preparation of stacking and resolving gel buffers

Stacking and resolving gel buffers were prepared by adjusting pH of 850 mL, 1.5 M Tris-base (Fisher Scientific) to 6.8 and 8.8, respectively. 6 M HCl was used for adjusting pH (Acros). The final volume was made up to 1 L. Millipore deionized (DI) water was used throughout the experiment.

### Preparation of running buffer

10X running buffer was prepared by dissolving 30 g Tris-base, 133 g Glycine (Sigma Aldrich) and 10 g Sodium dodecyl sulfate (SDS) (Omnipur) in 1 L water. For capillary electrophoresis, running buffer was diluted to 2X and for standard slab based PAGE, 1X concentration was used.

### Preparation of loading buffer

5X loading buffer was prepared by dissolving 302.4 mg of Tris-base, 770 mg Dithiothreitol (Promag), 1 g of SDS and 50% glycerol (Sigma Aldrich) in 10 ml water.

### Preparation of gel columns

General procedure for preparing gel columns was followed. Briefly, 12% resolving gel was prepared by mixing 3.33 ml water, 2.5 mL Tris-HCl buffer (pH-8.8), 4 mL mixture of acrylamide and Bis-acrylamide (Bio-Rad) in ratio 29:1, 100 µL of 10% SDS, 60 µL Ammonium persulphate (APS) (Sigma Aldrich) and 6.5 µL of Tetramethylethylenediamine (TEMED) (Fisher Scientific). 6% stacking gel was prepared by adding 700 µL mixture of acrylamide: Bis-acrylamide in ratio 29:1, 675 µL of Tris-HCl buffer (pH-6.8) and 50 µL of 10% SDS. To this 25 µL of APS and 20 µL of TEMED were added. For capillary (Fisherbrand) preparation, resolving gel columns of 1.5 or 2.5 cm length were made by injecting 30 or 50 µL of resolving gel solution into the capillary tube, respectively. This was followed by injecting 10 µL of stacking gel solution to form stacking gel of 0.5 cm length. The standard PAGE slab was prepared using the above mentioned solutions to form resolving and stacking gels of 4 cm and 1 cm length.

### Preparation and loading of samples

For loading 16 µL egg white, 16 µL water and 8 µL of loading buffer were pipette mixed to get uniform consistency. For capillary electrophoresis, 3 µL sample was loaded at the bottom of 500 µL centrifuge tube (Eppendorf) filled with the running buffer. This was immersed gently in the running buffer bottle with cathode. Then stacking gel side of the capillary tube was dipped into this such that the tip touches the sample. In this way, sample climbs the gel column against gravitational force purely due to the action of electrophoretic force. For standard PAGE, 5 µL of sample was placed in the wells created within stacking gel. PAGE was run by applying 180 V for 45 min.

### Staining of gels after electrophoresis

Standard PAGE gel slabs were stained using solution of 25 mg Coomassie Brilliant Blue (Sigma Aldrich) stain in 100 mL of methanol-acetic acid mixture (5:1). Gels were soaked in 10 mL of the above solution for 1 h under shaking condition. Later it was de-stained by placing the stained gel in methanol-acetic acid mixture (5:1) for 4 h.

## Electronic supplementary material

Supplementary Information
