# Supplementary material for: Electrophoresis assisted time-of-flow mass spectrometry using hollow nanomechanical resonators
Source: Sci Rep. 2017 Jun 14;7:3535. doi: 10.1038/s41598-017-03846-y (PMC5471201; doi:10.1038/s41598-017-03846-y)
Supplement: Supplementary file 1 — Supplementary Information [file 41598_2017_3846_MOESM1_ESM.doc]

Electrophoresis assisted time-of-flow mass spectrometry using hollow nanomechanical resonators

Swathi Chaudhari^1,$^, Kamalesh Chaudhari^1,$^, Seokbeom Kim^2^, Faheem Khan^1^, Jungchul Lee^2^,*, Thomas Thundat^1^,*

^1^Department of Chemical and Materials Engineering, University of Alberta, Edmonton, Alberta T6G 2V4, Canada.

^2^Department of Mechanical Engineering, 35 Baekbeom-ro (Sinsu-dong), Mapo-gu, Sogang University, Seoul, 04107, Korea.

^$^Equal contribution

### Correspondence and requests for materials should be addressed to T.T. and J.L. (email: [thundat@ualberta.ca](mailto:thundat@ualberta.ca), [jayclee@sogang.ac.kr](mailto:jayclee@sogang.ac.kr))

| **SI No.** | **Title** | **Page No.** |
| --- | --- | --- |
| Fig. S1 | Resonance frequency of the HNR as a function of the mass density of ethanol-water mixtures. | 3 |
| Fig. S2 | Sensitivity of the HNR to the molarity of BSA solution in deionized water and PAGE running buffer. | 4 |
| Fig. S3 | Raw data obtained after monitoring the resonance frequency of the HNR coupled to the outlet of capillary electrophoresis as shown in Fig. 1d. | 5 |
| Fig. S4 | Changes in the resonance frequency of the HNR when capillary electrophoresis was carried out by loading dye alone. | 6 |
| Fig. S5 | MALDI mass spectrum of egg white with the assignments for major protein peaks, their multiple charges and dimers. | 7 |
| Table S1 | Molecular weight and percentage of major proteins present in egg white. | 8 |

**Supplementary Information Figure S1**


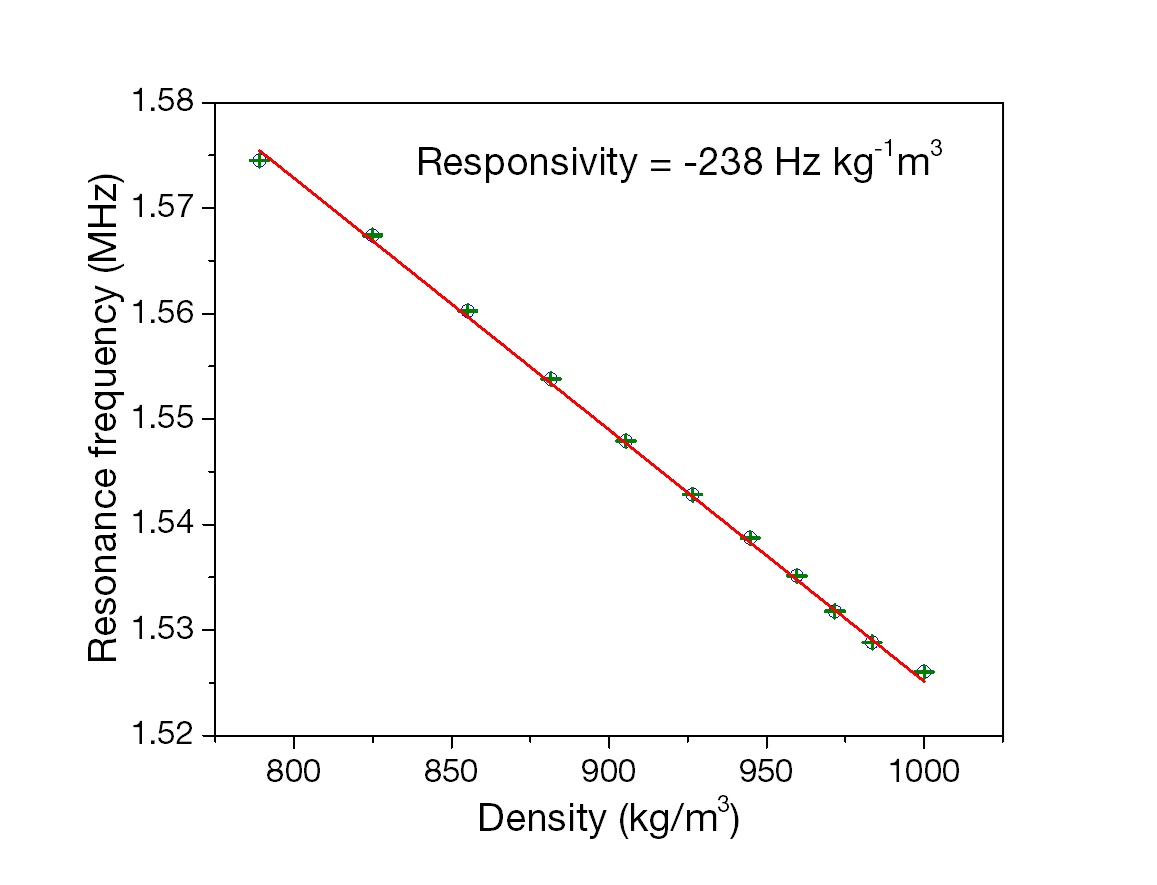


**Supplementary Figure S1** | Resonance frequency of the HNR as a function of the mass density of ethanol-water mixtures. Ethanol-water v/v ratio was varied from 0-9. The resonance frequency of the HNR was in linear relationship with the mass density of solution loaded into its integrated channels. The mass responsivity was −238 Hz kg^-1^ m^3^.

**Supplementary Information Figure S2**


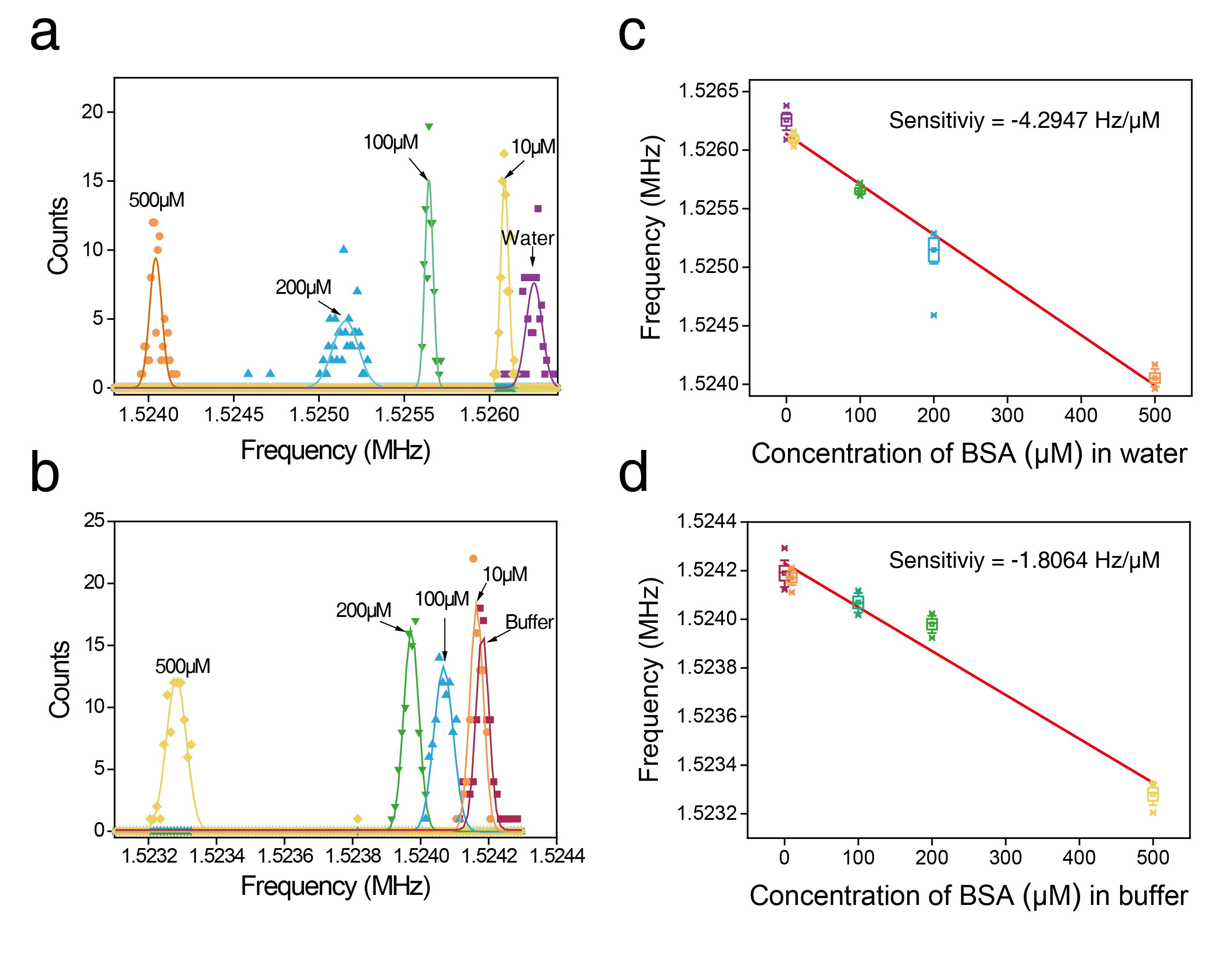


**Supplementary Figure S2 |** Sensitivity of the HNR to the molarity of BSA solution in deionized water and PAGE running buffer (a-b) Gaussian curve fitting to the resonance frequency distributions of the HNR when it was loaded with the solution of BSA in deionized water (a) or in a running buffer of PAGE (b). Molarity of solution was varied from 0 to 500 µM. (c-d) The resonance frequency of the HNR follows linear trends with the change in the molarity of BSA solution. The responsivity of HNR was −4.2947 Hz µM^-1^ for solutions of BSA in DI water (c) and −1.8064 Hz µM^-1^ for solutions of BSA in a running buffer of PAGE (d), respectively (Boxes and whiskers in the plot show standard deviation, and 5 % and 95 % values, respectively. Symbols show minimum and maximum values. 90 samples were measured for each points).

**Supplementary Information Figure S3**


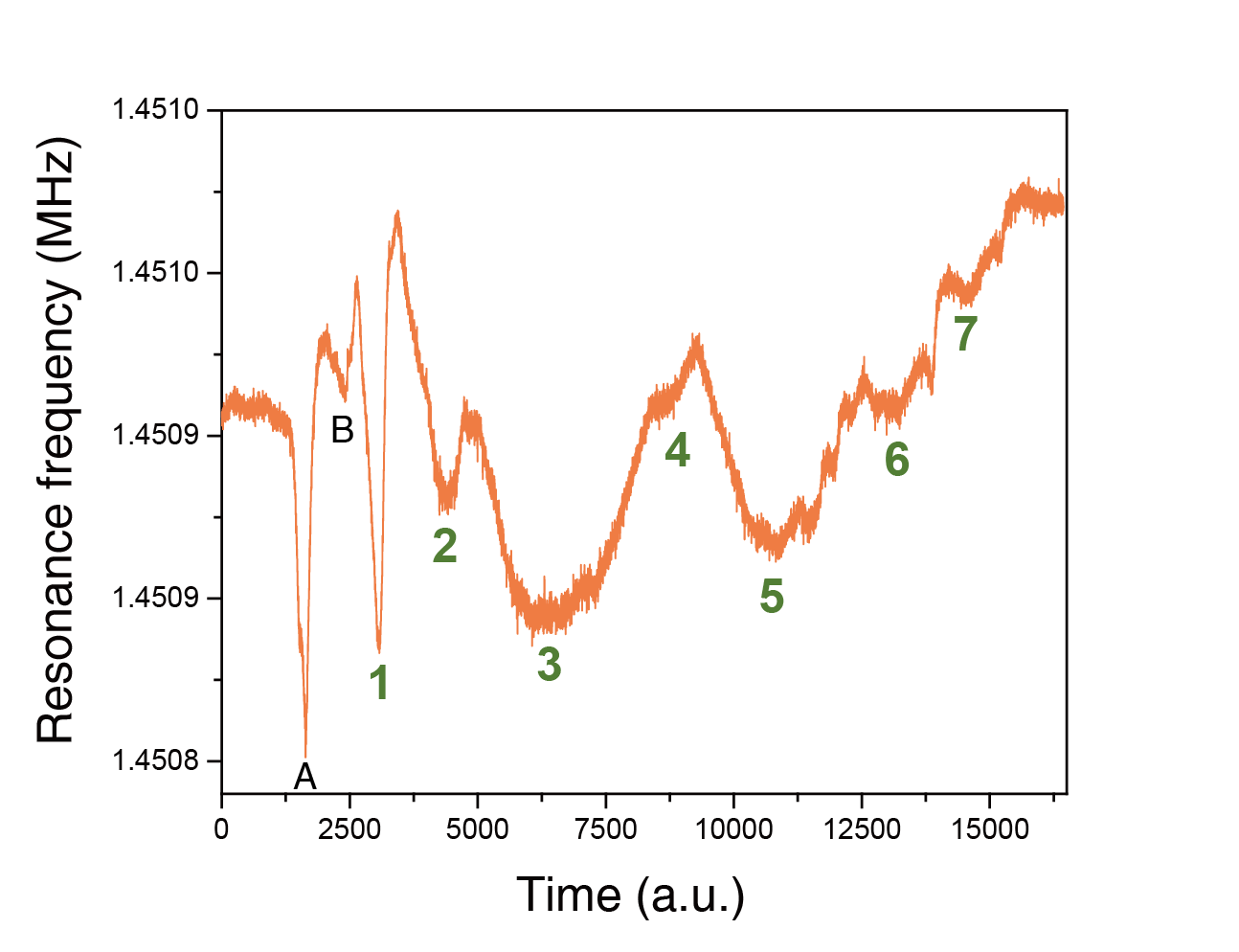


**Supplementary Figure S3 |** Raw data obtained after monitoring the resonance frequency of the HNR coupled to the outlet of the capillary electrophoresis tube, as shown in Fig. 1d.

Fig. S3 was processed further to obtain the mass spectrum shown in Fig. 2b. Data processing was performed in following steps,

1. Data was normalized to get minimum value 0 and maximum value 1.

2. Value of -1 was subtracted from data and then it was multiplied by -1 to prepare the data for peak fitting.

3. Then peak fitting and baseline correction was performed using Gaussian distribution function.

The dips A and B appear as a result of dye front which can be observed when experiment was carried out in the absence of a protein sample. This baseline data is shown in Fig. S4.

**Supplementary Information Figure S4**


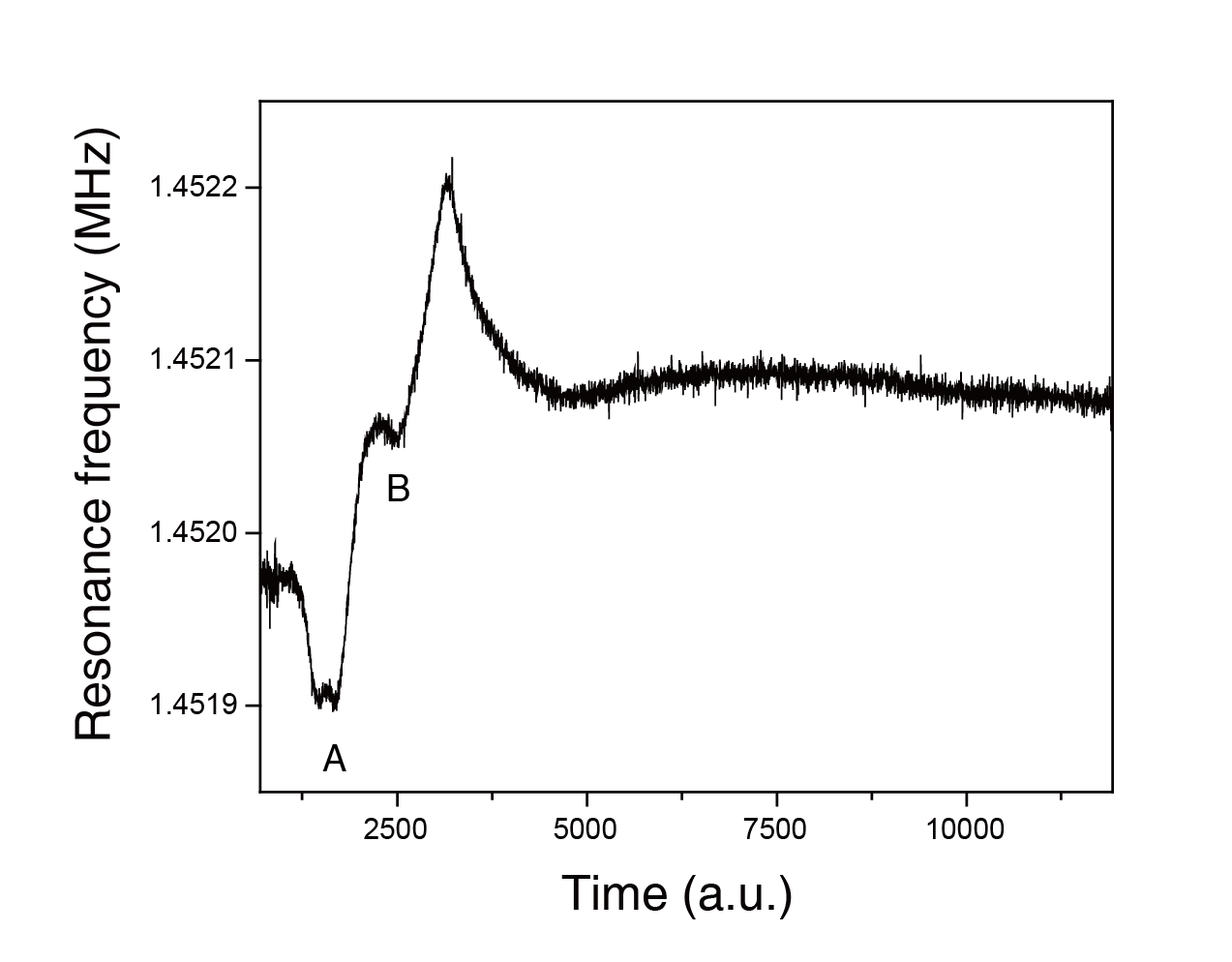


**Supplementary Figure S4 |** Changes in the resonance frequency of the HNR when capillary electrophoresis was carried out by loading dye alone.

The curve in Fig. S4 shows two dips (labeled as A and B) which are assigned to dye front in the mass spectrum of egg white. After these two dips, baseline of temporal resonance frequency curve shifts towards higher frequency and does not come to original value unless device is thoroughly rinsed with deionized water. This is possibly due to the continuous charge flow which induces heating of the resonator.

**Supplementary Information Figure S5**

**Supplementary Figure S5 |** MALDI mass spectrum of egg white with the assignments for major protein peaks, their multiple charges and dimers.

MALDI mass spectrum data was collected using Applied Biosystems Voyager DE Pro MALDI MS instrument. A pulsed nitrogen laser of 337 nm was used for ionization. Positive-ion mode mass spectrum was collected and averaged for 100-250 shots. Sample preparation was done using a previously reported protocol by Chaudhari *et al*.^1^.

**Supplementary Information Table S1**

**Table S1.** Molecular weight and percentage of major proteins present in egg white^2^.

**References**

1. Chaudhari, K., Xavier, P.L., Pradeep, T. Understanding the evolution of luminescent gold quantum clusters in protein templates. *ACS Nano*. **5,** 8816-8827 (2011)

2. Yamamoto, T., Juneja, L. R., Hatta, H., Kim, M. Hen Eggs: Basic and Applied Science (ed. Tamamota, T.) 19, (CRC press, 1996).
